# The standard operating procedure of the DOE-JGI Metagenome Annotation Pipeline (MAP v.4)

**DOI:** 10.1186/s40793-016-0138-x

**Published:** 2016-02-24

**Authors:** Marcel Huntemann, Natalia N. Ivanova, Konstantinos Mavromatis, H. James Tripp, David Paez-Espino, Kristin Tennessen, Krishnaveni Palaniappan, Ernest Szeto, Manoj Pillay, I-Min A. Chen, Amrita Pati, Torben Nielsen, Victor M. Markowitz, Nikos C. Kyrpides

**Affiliations:** Genome Biology Program, Department of Energy Joint Genome Institute, 2800 Mitchell Drive, Walnut, Creek USA; Biosciences Computing, Computational Research Division, Lawrence Berkeley National Laboratory, 1 Cyclotron Road, Berkeley, USA

**Keywords:** Metagenome annotation, SOP, IMG, JGI

## Abstract

The DOE-JGI Metagenome Annotation Pipeline (MAP v.4) performs structural and functional annotation for metagenomic sequences that are submitted to the Integrated Microbial Genomes with Microbiomes (IMG/M) system for comparative analysis. The pipeline runs on nucleotide sequences provided via the IMG submission site. Users must first define their analysis projects in GOLD and then submit the associated sequence datasets consisting of scaffolds/contigs with optional coverage information and/or unassembled reads in fasta and fastq file formats. The MAP processing consists of feature prediction including identification of protein-coding genes, non-coding RNAs and regulatory RNAs, as well as CRISPR elements. Structural annotation is followed by functional annotation including assignment of protein product names and connection to various protein family databases.

## Introduction

The DOE-JGI Metagenome Annotation Pipeline (MAP) supports the structural and functional annotation of metagenomic datasets submitted to the Integrated Microbial Genomes with Microbiomes (IMG/M) system [1]. The annotation includes the prediction of CRISPR elements, non-coding and protein-coding genes, and ends with the assignment of a product name and the prediction of functions for each gene. The annotated metagenomic datasets produced by MAP are integrated into IMG/M where they can be analyzed or revised in the context of a comprehensive set of publicly available genomes and metagenomes.

The DOE-JGI MAP requires a multi-FASTA file of assembled nucleotide sequences and/or a fastq file containing unassembled 454, Illumina or PacBio reads as input, though no assembly is performed on the unassembled reads. To submit sequence datasets for annotation they need to be linked with an analysis project that previously has been specified in the Genomes OnLine Database [2].

Annotation of metagenomic sequences in MAP is organized in three stages: sequence data pre-processing, structural annotation, functional annotation and phylogenetic lineage prediction for scaffolds/contigs. Identification of genes and repeats produces a GFF file without any functional information for the predicted genes. These protein coding genes are then assigned with a function followed by integration into IMG.

## Procedure and implementation

The MAP stages and individual steps are further described below. All tools, parameters and cutoffs are the same for assembled and unassembled sequences, unless otherwise stated.

### Sequence data pre-processing

**Fig. 1:**
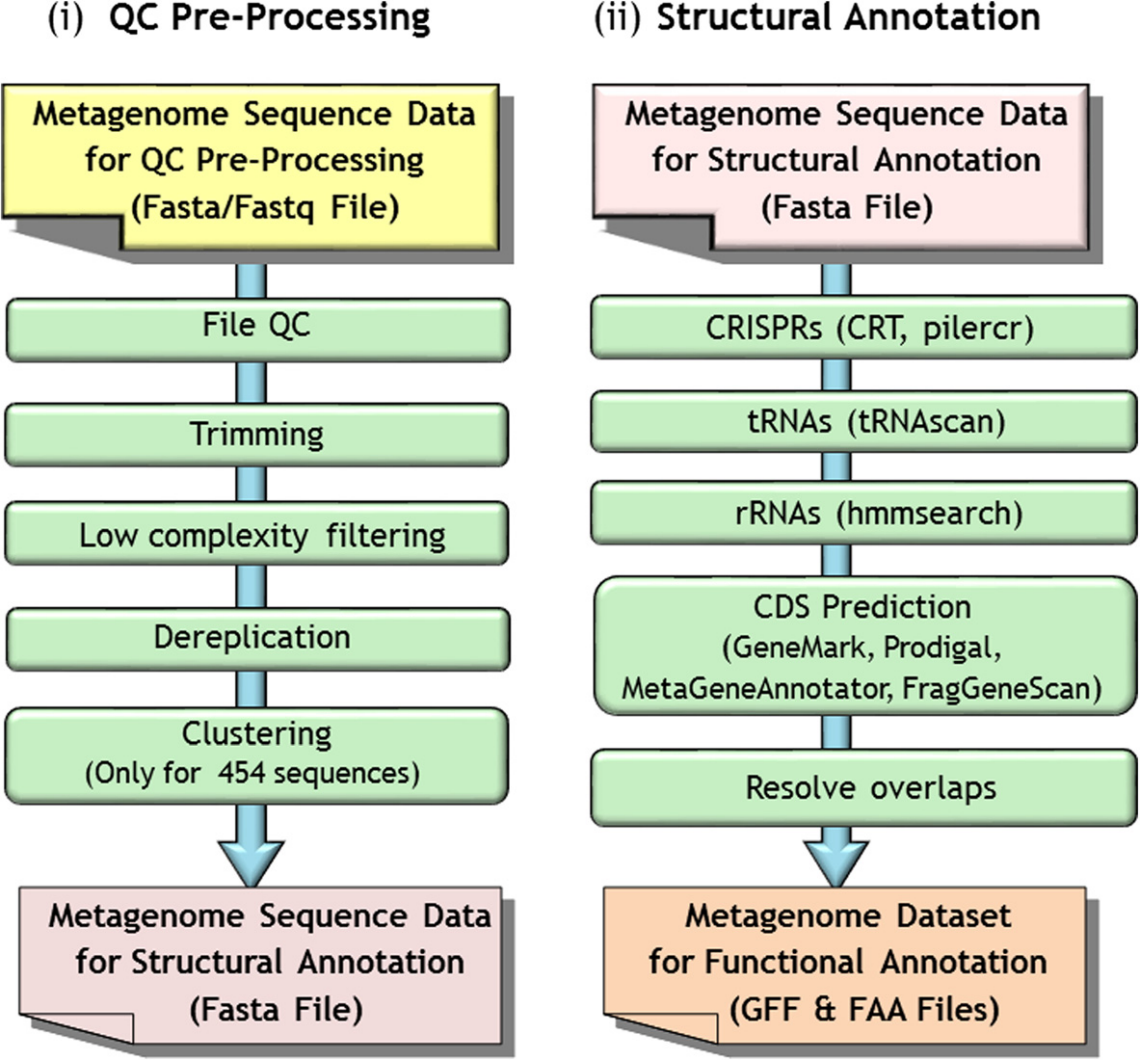
Metagenome sequence data pre-processing and structural annotation steps of the MAP v.4

In order to reduce noise and eliminate low-quality and low-complexity sequences, such as oligomers of sequencing primers and adaptors, a preprocessing step is implemented for all the metagenomic datasets (Fig. 1(i)). First, ambiguous nucleotides in the sequence datasets are replaced by N’s, while sequences having any characters other than {A,C,G,T,N} are removed from further processing. Additionally, all sequences are renamed in order to ensure that there are no duplicate sequence names and the names comply with the requirements of all the tools employed in subsequent stages. The pipeline creates a file that maps the old sequence names to the new ones.

For fastq files with unassembled reads a check for the matching lengths of each sequence and its quality values is applied. Sequences shorter than 150 bp are removed; unassembled 454 reads longer than 1000 bp are also removed.

Second, the sequences are trimmed in order to remove low-quality regions and trailing ‘N’s. In the case of unassembled reads, quality data from fastq files is used with Lucy 1.20 [3] with a threshold of Q13 for Illumina reads and Q20 for 454 reads in order to identify and trim regions of low-quality at the ends of the reads. In the case of Illumina reads the longest contiguous sub-sequence that passes the Q13 threshold for all residues is retained. Unassembled 454 and Illumina reads containing more than five occurrences of ‘N’s are removed. Sequences shorter than 150 bp after trimming are also removed. The trimmed sequences then go through a low complexity filtering where sequences containing low complexity regions are identified and removed using dustmasker 1.0.0 [4].

For unassembled 454 reads, MAP performs a de-replication step to remove replicated sequences shown to be an artifact of the 454 pyrosequencing technique [5]. When two or more sequences are at least 95 % identical, with their first 3 bps being identical as well, those sequences are considered to be replicates and only the longer copy is retained. For unassembled Illumina reads, the same method is used to reduce the dataset size, except that the first 5 bps need to be identical for two sequences to be considered as replicates.

To further reduce the 454 dataset size we execute an additional clustering step. Due to the 454 homopolymer issue [6], unassembled 454 reads are clustered by first splitting each sequence in kmers, with each kmer reduced to a non-tandem sequence kmer, and then comparing if two sequences have identical kmers (DeClust 1.0, Mavromatis K, unpublished).

### Structural annotation

Scaffolds that have stretches of 50 Ns or more are separated into contigs in order to facilitate gene prediction. Scaffolding information is retained and contigs are assembled back into scaffolds after structural annotation. The first step in feature prediction is the identification of CRISPRs and non-coding RNA genes (tRNA, rRNA and other RNA genes), followed by prediction of protein coding genes, as shown in Fig. 1(ii).

Identification of CRISPR elements is performed using the CRT [7] and PILER-CR v1.06 [8] tools. The PILER-CR settings include maximum spacer length of 100 bp and the CRISPR element is required to have at least 5 repeats, which need to have at least 90 % identity with each other and 75 % identity with the consensus sequence. The MAP pipeline also runs a modified version of the CRT-CLI 1.2 version. The modified CRT has the capability to read multi-FASTA files, detect truncated repeats at the ends of the contigs/scaffolds as well as the anchor repeat in the trail end and deal with spacer artifacts and repeats that contain Ns. This version also executes checks for repeat and spacer length ratios, while the length and similarity checks are performed as part of “all vs. all” spacer and repeat comparisons. Furthermore, the progression step of the sliding search window is reduced to 1, while threshold values and search ranges, which are strictly defined in the original software, can be changed from default values on the command line together with the new options and arguments. In the CRT version implemented in the MAP pipeline, the default values for the minimum and maximum repeat lengths are set to 20 and 50 bp, respectively, while the minimum and maximum spacer lengths are set to 20 and 60 bp, respectively. The ratio of the spacer lengths to the repeat lengths are required to be between 0.6 and 2.5. The default search window is 7 bp long and an element needs to have at least 3 repeats that have a minimum of 70 % identity. The predictions from PILER-CR and CRT are concatenated and when overlapping, the CRT predictions are retained.

Protein-coding genes and non-coding RNA genes are identified using a combination of Hidden Markov Models and *ab initio* gene callers. The first category of non-coding RNAs, tRNAs, are predicted using tRNAscan SE-1.3.1 [9] which requires the domain of the organism (*Bacteria*, *Archaea,**Eukaryota*) as a parameter. A metagenome is a potential mixture of the three domains of life, so the program is run for each domain, that is three times, with the best scoring predictions selected.

Ribosomal RNA genes (5S, 16S, 23S) are predicted using hmmsearch tool from the package HMMER 3.1b2 [10]. Since the domain is a parameter that is required for rRNA prediction, the pipeline runs it again three times against in-house curated models, derived from full length genes within IMG, while keeping the best scoring models.

The identification of protein-coding genes is performed using a consensus of four different *ab initio* gene prediction tools: prokaryotic GeneMark.hmm (v. 2.8) [11], MetaGeneAnnotator (v. Aug 2008) [12], Prodigal (v. 2.6.2) [13] and FragGeneScan (v. 1.16) [14]. The predictions from all the tools are combined and protein-coding genes with translations shorter than 32 amino acids are deleted. A majority rule-based decision schema is then followed in order to select gene calls. When there is a tie between two or more different gene models, selection is based on the preference order of gene callers determined by benchmarking of the individual gene finders on simulated metagenomic datasets (GeneMark > Prodigal > MetaGeneAnnotator > FragGeneScan). Overlaps between predicted features of different type are resolved as follows:

- For conflicts between rRNA and protein-coding genes, a protein-coding gene completely encompassed by rRNA is deleted. In the case of the protein-coding gene partially or completely overlapping the rRNA, an attempt is made to identify an alternative start site for the protein-coding gene to remove the overlap. If that fails the protein-coding gene gets deleted.
- For tRNA and rRNA conflicts the lower scoring prediction is deleted.
- For tRNA and protein-coding gene conflicts a check is executed if the protein-coding gene has a hit to a Pfam. If there is a hit both predictions are kept, otherwise the conflict is resolved using the same rules as for the rRNA and protein-coding gene conflict.
- CRISPR element and protein-coding gene conflicts are resolved following the rule for the rRNA and protein-coding gene conflicts.

Every annotated gene is assigned a locus tag of the form PREFIX_#####, where the prefix is the identifier of the GOLD Analysis Project associated with the metagenome dataset. The first “#” indicates the sequence type: 1 = assembled, 2 = unassembled 454 sequence, 3 = unassembled Illumina sequence, 4 = unassembled PacBio sequence. It is followed by one or more digits indicating the sequence number within the dataset and the number of the gene on this particular sequence (which gets incremented by one for each following gene). Thereby each locus tag provides a unique identifier for every gene within a sequencing project.

The output of this stage consists of two files: a fasta formatted file containing all CDS protein sequences and a GFF formatted file placing predicted features on the metagenome sequences.

**Fig. 2:**
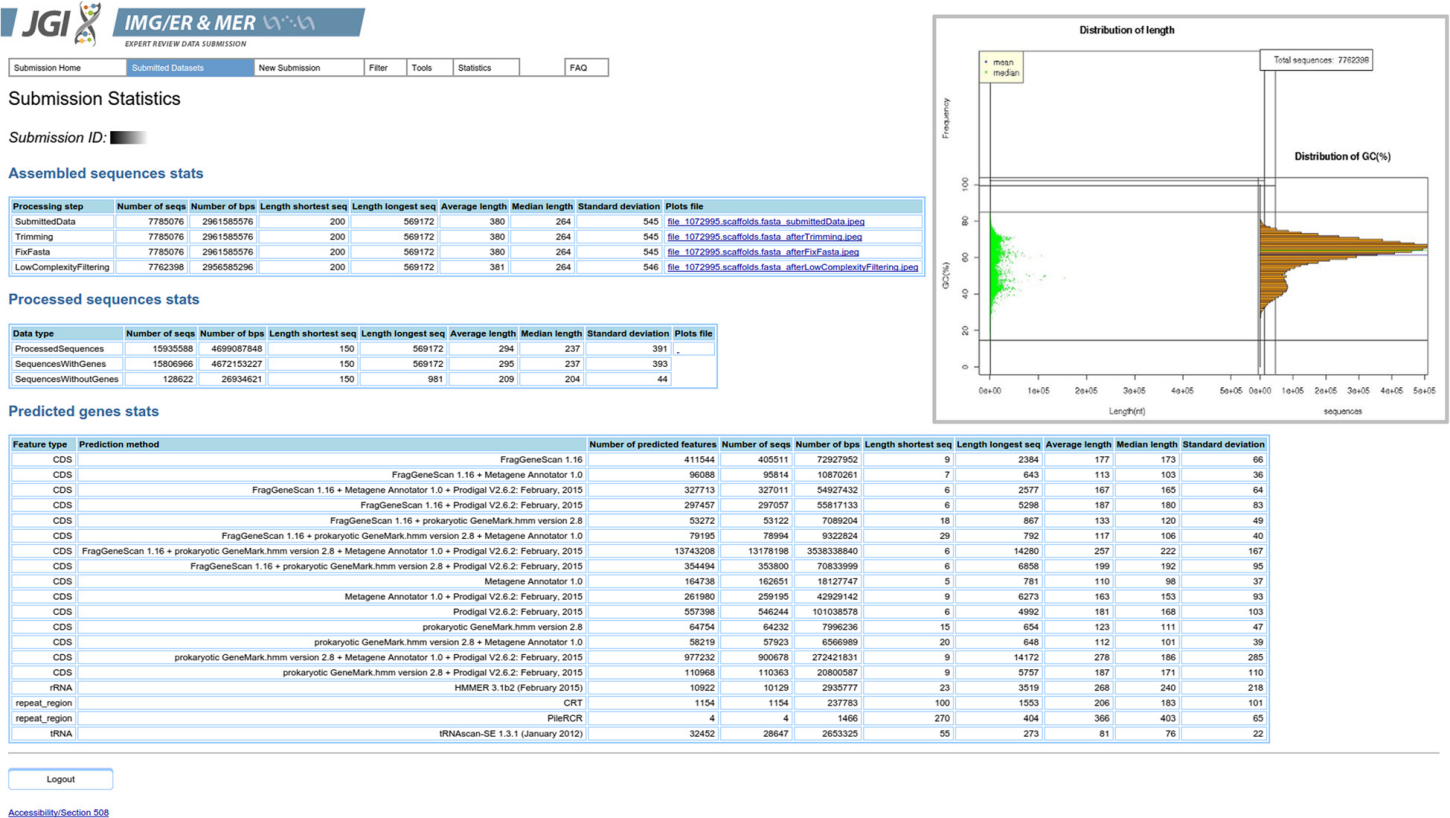
Submission statistics created by the MAP v.4

After the pre-processing and the structural annotation completed successfully, basic statistics, e.g. number of sequences, sequence lengths distribution and number of genes predicted by each tool, can be viewed on the details page of every submission (Fig. 2).

### Functional annotation

Functional annotation for metagenomes consists of associating protein-coding genes with COGs, Pfams, KO terms, EC numbers and phylogenetic lineage for scaffolds/contigs.

Genes are associated with COGs by comparing protein sequences to COG PSSMs from the CDD database [15], using RPS-BLAST 2.2.31 with an e-value of 10e-2 (0.1). If the overlap between two COG predictions is greater than half of the length of the shorter model, the hit having the largest bit score, lowest e-value, longer alignment length or higher percent identity, is retained.

Genes are associated with Pfam-A by comparing protein sequences to the Pfam database [16] using HMMER 3.1b2. Model specific trusted cut-offs are used with hmmsearch (--cut_tc), with output filtering following the same rules as mentioned above for COG assignments.

Genes are associated with KO terms [17] and EC numbers based on USEARCH 6.0.294 results [18] comparing metagenome proteins against an isolate genome reference database with maxhits of 50 and an e-value of 0.1. An isolate genome reference database is assembled using all non-redundant protein sequences from public, high quality genomes in IMG. The top 5 hits to genes in the KO index are used, with an assignment made only if there is at least 30 % identity and at least 70 % of the KO gene sequence is covered by the alignment. EC number assignments are derived from KO assignments using KEGG KO to EC mapping. One top USEARCH hit per gene is also retained for the Phylogenetic Distribution tool in IMG and assignment of phylogenetic lineage to scaffolds and contigs. The latter is assigned as the last common ancestor of USEARCH hits of the genes on the scaffold/contig provided that at least 30 % of the genes have USEARCH hits.

Optional scaffold/contig coverage information, if provided by the user at the time of the submission, is used to calculate “estimated gene copies”, whereby the number of genes is multiplied by the average coverage of the contigs, on which these genes were predicted. This feature is important for accurate estimation of abundance of protein families, such as COGs, Pfams or KO terms, and phylogenetic lineages found in the metagenomes with assembled scaffolds/contigs, which collapse many unassembled reads into a single sequence. In the absence of coverage information most abundant and well-assembled phylogenetic lineages and protein families may appear underrepresented in the abundance analyses.

### Product name assignment

Protein product names are assigned based on the name of their associated protein families, as follows:

1. If the gene has a COG assigned, the gene has at least 20 % identity to the COG PSSM, and the alignment length is at least 70 % of the COG consensus length, then the COG name is assigned as product name. If the COG name is “uncharacterized conserved protein” or contains “predicted”, the name has the format “COG name - COG ID”. If either the percent identity or alignment length condition is not satisfied, a check whether the COG and any Pfams assigned to the gene are found in a COG-Pfam correspondence table. This table has been generated by mapping COGs onto Pfams through the genes to which both are assigned. If at least one of the gene’s Pfams matches the respective COG in COG-Pfam correspondence table, the COG name is assigned as product name, even though the percent identity and alignment length for COG hit does not satisfy the above criteria.
2. For genes that were not associated with a product name using COG, product names are assigned based on the name of their associated Pfam, if a gene has at least one Pfam assigned to it.

### Functional annotation sources

- COG 2014, November 2014
- KEGG Release 71.0, July 2014
- PFAM 28.0, May 2015
- IMG NR, September 2015

## Discussion

The MAP pipeline provides rapid automatic annotation of metagenome datasets. It is largely based on publicly available software supplemented with custom scripts for data handling and seamless integration of the input and output of different programs. The functional annotation is implemented within the Hadoop framework (https://hadoop.apache.org/). Consistency and reproducibility of the results produced by MAP depend on the databases and software used in the pipeline. New, updated versions of databases like Pfam and KEGG allow the prediction of more genes and more precise annotations. The pipeline is publicly available to the genomics community who can annotate their dataset by submitting them via the IMG submission site [1]. We will continue to improve the MAP pipeline by extending the existing software and adding new tools that allow the identification and characterization of more features in the metagenome datasets. Additionally, we will be working on porting compute-intensive steps, e.g. the functional annotation, to the supercomputers located at the National Energy Research Scientific Computer Center (NERSC), such as Edison and Cori.
